# Demonstration of nearly pinhole-free epitaxial aluminum thin films by sputter beam epitaxy

**DOI:** 10.1038/s41598-020-74981-2

**Published:** 2020-10-27

**Authors:** Ka Ming Law, Sujan Budhathoki, Smriti Ranjit, Franziska Martin, Arashdeep S. Thind, Rohan Mishra, Adam J. Hauser

**Affiliations:** 1 Department of Physics and Astronomy, The University of Alabama, Tuscaloosa, AL 35487 USA; 2 Institute of Materials Science and Engineering, Washington University in St. Louis, St. Louis, MO 63130 USA; 3 Department of Mechanical Engineering and Materials Science, Washington University in St. Louis, St. Louis, MO 63130 USA

**Keywords:** Materials for devices, Electronic devices, Information storage, Materials science, Design, synthesis and processing

## Abstract

Superconducting resonators with high quality factors have been fabricated from aluminum films, suggesting potential applications in quantum computing. Improvement of thin film crystal quality and removal of void and pinhole defects will improve quality factor and functional yield. Epitaxial aluminum films with superb crystallinity, high surface smoothness, and interface sharpness were successfully grown on the c-plane of sapphire using sputter beam epitaxy. This study assesses the effects of varying substrate preparation conditions and growth and prebake temperatures on crystallinity and smoothness. X-ray diffraction and reflectivity measurements yield extensive Laue oscillations and Kiessig thickness fringes for films grown at 200 °C under 15 mTorr Ar, indicating excellent crystallinity and surface smoothness; moreover, an additional substrate preparation procedure which involves (1) a modified substrate cleaning procedure and (2) prebake at 700 °C in 20 mTorr O_2_ is shown by atomic force microscopy to yield nearly pinhole-free film growth while maintaining epitaxy and high crystal quality. The modified cleaning procedure is environmentally friendly and eliminates the acid etch steps common to conventional sapphire preparation, suggesting potential industrial application both on standard epitaxial and patterned surface sapphire substrates.

## Introduction

Superconducting resonators have attracted attention due to their applications in quantum computing and microwave detection^1^. Patterning of superconducting resonators typically involves photolithography, ion milling, or chemical wet etch processes on an epitaxial thin film deposited onto a carefully chosen substrate. Sapphire is a strong choice for superconducting applications as large (up to 30 cm in diameter) sapphire single crystals with low twinning can be manufactured easily using the Czochralski method; crystal uniformity promotes dielectric homogeneity, which is required from substrates for superconducting resonators or general superconductor-based integrated circuits^2^. In addition, choosing a substrate with a low dielectric loss prevents the quality factor Q of an ideal resonator structure from being limited by the dielectric loss of the chosen substrate:

$${Q}^{-1}=\mathrm{tan}({\delta }_{subs})+\mathrm{tan}\left({\delta }_{film}\right)$$

where the first and second terms are the dielectric loss tangents of the substrate and superconducting film, respectively. Sapphire has one of the lowest dielectric loss values known, with reported values as low as 10^–7^ to 10^–8^, versus values of 10^–6^ to 10^–7^ for typical substrate materials^3,4^. In addition, processes to create atomically smooth surfaces are well-known and aid high-quality film growth^5^. Low loss, high mechanical strength, low thermal expansion coefficient, and chemical inertness make sapphire a compelling substrate choice for superconducting resonators.

Much work has been done on superconductivity of aluminum thin films on sapphire^6–12^, and a number of successfully prepared superconducting Josephson tunnel junctions from aluminum are now being used as the basis for quantum bit designs^13–17^. Fabrication of superconducting resonators from aluminum films on sapphire is advantageous due to the low cost of both aluminum and the high quality of sapphire. Sapphire substrates also have low lattice mismatch with aluminum, allowing higher film quality without the need for elaborate substrate preparation and time-consuming growth procedures^6^. Higher film quality leads to a lower film dielectric loss tangent, thus a higher quality factor^2^. However, unexpected losses in such superconducting resonators have been observed and are theorized to originate from two-level system (TLS) defects, commonly a result of poor metal-substrate interface, crystallinity, and film surface quality^18–24^. As such, improvement in interfacial crystal quality is required for superconductive loss reduction and improved device performance.

Epitaxial aluminum films also hold significant attention in plasmonics due to their low intrinsic loss in the UV regime compared to Au and Ag, a narrower energy range where inter-band transitions are active^25–31^, and their compatibility with current CMOS technology^27–29^. UV metal–oxide–semiconductor (MOS) nanolasers that consist of ZnO nanowires deposited on epitaxial aluminum films feature low lasing thresholds, high characteristic temperatures, and can even be operated at room temperature^25–28^. In addition, surface aluminum oxide can either act as a natural anodization for the film or be exploited for its dielectric properties, as in the case of Al–Al_2_O_3_ nanodisks^31–33^.

This study demonstrates epitaxial growth of an aluminum thin film on c-plane sapphire using DC magnetron sputtering. By (1) tuning the growth temperature and pressure, (2) performing our modified substrate cleaning procedure, and (3) prebaking substrate in an oxygen-rich environment before film growth, films with a superior combination of high smoothness and crystallinity, and with an extremely low density of surface pinhole defects, have been prepared.

## Methods

Films were fabricated using an ultra-high-vacuum (UHV), off-axis, combinatorial, DC magnetron sputter beam epitaxy system made by AJA International Inc., with beam-shaping shutter control and growth rate tuning via quartz crystal microbalance (QCM). Simultaneous use of four concentrically arranged aluminum targets (99.999% purity) provided the best results with a system base pressure of ~ 10^–9^ mTorr and substrate rotation. All films were sputtered in the presence of Ar gas, on whole or cut c-plane sapphire 2″ wafers, after being prebaked in a vacuum chamber under a range of temperatures and O_2_ pressures.

Structural characterization to determine crystal quality was performed by off-axis x-ray diffraction (XRD) and x-ray reflectivity (XRR) using a Philips X’Pert diffractometer using a Cu anode with K_β_ filter. Since Cu Kα is a doublet excitation, single diffraction peaks in XRD patterns in this paper may appear as twin peaks; although, the doublet nature of observed peaks is easily identifiable and does not pose a difficulty in our x-ray analysis. In addition, film surface roughness characterization was performed with tapping-mode atomic force microscopy (AFM) using Digital Instruments Dimension 3100 with Nanosensors Point-Probe-Plus cantilevers.

Scanning transmission electron microscopy (STEM) imaging and electron energy loss spectroscopy (EELS) were carried out using the Nion UltraSTEM 200 microscope (operating at 200 kV) at Oak Ridge National Laboratory. The microscope is equipped with a fifth-order aberration corrector and a cold-field emission gun. The cross-section sample for STEM characterization was prepared by parallel polishing using a MultiPrep system followed by low angle ion milling. The ion milling was carried out at 5 kV followed by final polishing at 2 kV and 1 kV, using a Fischione model 1010 low angle ion milling and polishing system. The cross-section sample was baked at 160 °C under vacuum for at least 8 h prior to the STEM experiments to minimize contamination. EELS data acquisition was carried out using a Gatan Enfinium spectrometer, with a collection semi-angle of 33 mrad, an energy dispersion of 0.5 eV per channel, and pixel dwell time of 0.5 s. To remove random-noise components from EELS elemental maps, we have performed principal component analysis (PCA). Elemental maps were obtained by integrating the core-loss signal for each element after subtracting the background signal using a power law prior to the core-loss edge.

## Optimization and results

**Figure 1:**
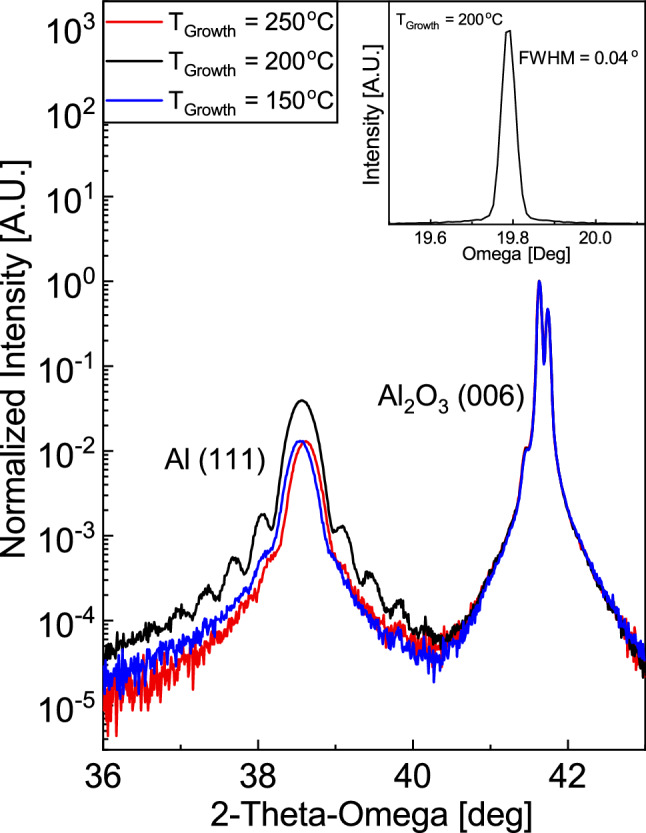
2θ-ω XRD patterns of Al films on Al_2_O_3_ (001) substrates, with varying growth temperature. Al (111) peak at 38.5°, Al_2_O_3_ (006) substrate peak at 41.6°. Inset: rocking curve for sample grown at 200 °C.

First, an optimal growth temperature for aluminum on c-plane sapphire was identified. Substrates were loaded into the sputter chamber and prebaked in vacuum at 500 °C for 30 min to remove water and organic contaminants. After prebake, the substrate was cooled in vacuum to growth temperatures of 150 °C, 200 °C, or 250 °C, and films were grown in 5 mTorr Ar gas environment. Figure 1 shows X-ray diffraction (XRD) patterns for films grown at 150 °C, 200 °C, and 250 °C, respectively. All three samples exhibit clear Al_2_O_3_ (006) peaks and Al (111) peaks. However, only the Al (111) peak from the 200 °C sample features Laue diffraction oscillations, indicative of atomically smooth substrate-metal and metal-vacuum interfaces. The rocking curve on the Al (111) peak has a full-width at half maximum (FWHM) of 0.040°, which is comparable or better than those grown by molecular beam epitaxy and reported elsewhere^28,34^. Moreover, we report clear Laue oscillations on either side of the Al (111) peak in Fig. 1, which have not been reported previously. Our sample series found an optical growth temperature of 200 °C.

**Figure 2:**
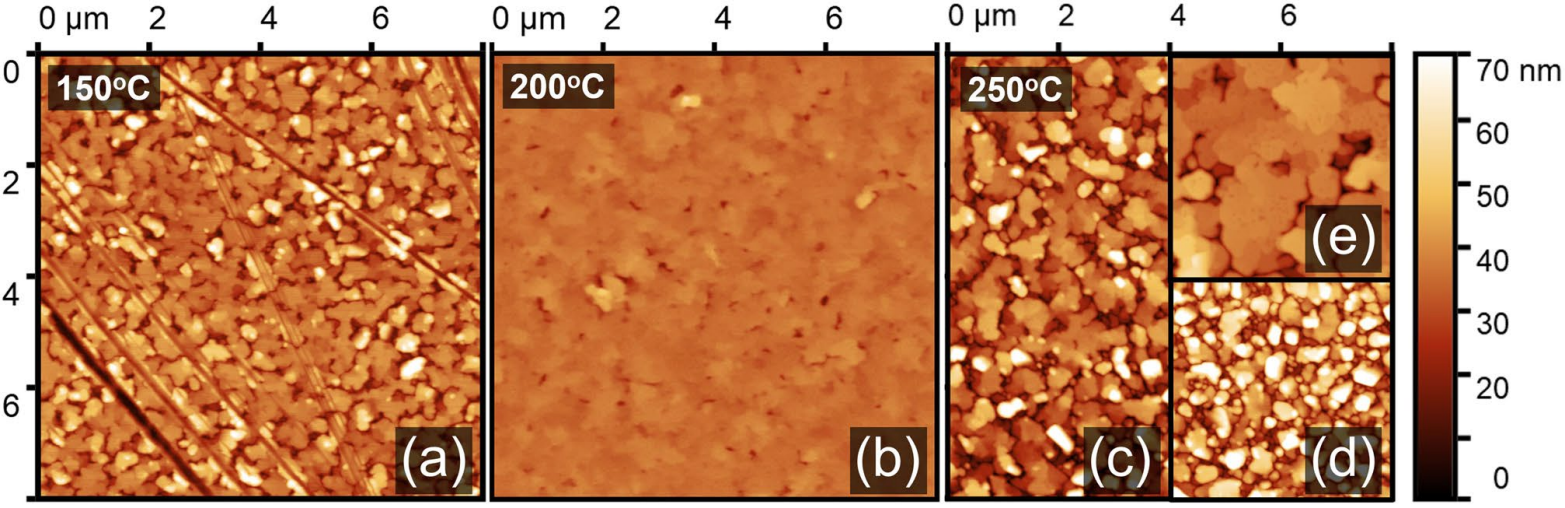
AFM images of Al/Al_2_O_3_ grown at (**a**) 150 °C, (**b**) 200 °C, and (**c**) 250 °C. The sample grown at 250 °C exhibits regions of particulate growth (**d**) and smooth regions (**e**). The scratch marks seen in the 150 °C sample were caused by post-growth handling.

Figure 2 shows atomic-force microscopy (AFM) images for samples grown at 150 °C, 200 °C, and 250 °C, respectively. The trend in surface roughness is consistent with that suggested by XRD, in that the 200 °C sample shows a significantly smoother surface than the other samples. The 150 °C sample features a surface with partially merging grains with short valley terrains; the 200 °C sample surface appears to be transitioning away from this initial 3D growth as grains begin to merge to form a smooth surface; the 250 °C sample has dense particulates in localized regions of at least 12 μm in diameter, indicating that 3D growth resurfaces as particulates start to nucleate.

**Figure 3:**
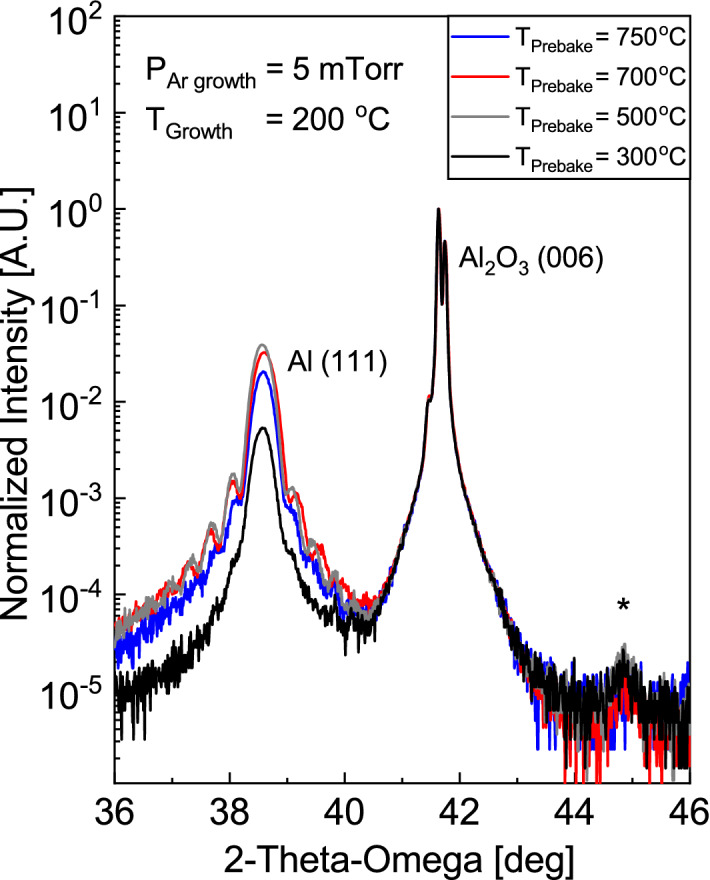
2θ-ω XRD patterns of Al films on Al_2_O_3_ (001) substrates, with varying prebake temperature. Al (111) peak at 38.5°, Al_2_O_3_ (006) substrate peak at 41.6°. Note the defect peak at 44.9°.

Previous work has shown that substrate prebake in vacuum at high temperatures leads to removal of surface contaminants and hydroxides, and yields aluminum films with better surface smoothness, uniformity, and crystallinity^35^. Optimization by prebake procedures was explored on samples grown at our optimal conditions (200 °C, P_Ar_ = 5 mTorr) after prebake heating at 300 °C, 500 °C, 700 °C, or 750 °C for 30 min in vacuum. After prebake, substrates were cooled under vacuum until the growth temperature of 200 °C was reached, at which point film deposition was begun. Figure 3 shows XRD patterns for samples prepared with different prebake temperatures. Note that all samples featured in this series exhibited a small Al (200) peak; since this orientation does not form an epitaxy with the substrate nor with Al (111), we suspect that it originates from an undesired secondary phase related to the early formation of faceted voids. Micron-sized faceted voids have been observed previously and postulated to be caused by substrate surface defects^36^. All samples feature Laue diffraction fringes on the Al (111) peak, but those prebaked at 500 °C and 700 °C have the most prominent and extensive fringes, indicating best surface/interface quality. As the same samples also have the largest Al (111) peak intensities, we conclude that a vacuum prebake temperature of 500–700 °C results in the best combination of film crystallinity and surface quality.

**Figure 4:**
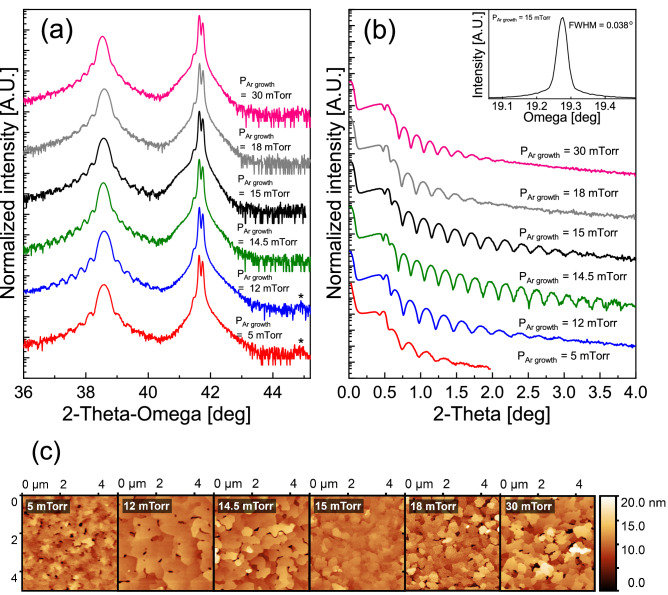
With varying growth pressure: (**a**) XRD; (**b**) XRR thickness (Kiessig) fringes and rocking curve (inset) for sample grown at 15mTorr; (**c**) AFM images (left to right: 5, 12, 14.5, 15, 18, 30 mTorr).

After determining optimal growth and prebake temperatures, the effect of growth pressure was examined. Six Al films were grown at 200 °C under 5 mTorr, 12 mTorr, 14.5 mTorr, 15 mTorr, 18 mTorr, and 30 mTorr Ar pressure, respectively, all with the substrate having been prebaked at 700 °C for 30 min under vacuum. Figure 4a shows the XRD patterns for the samples grown at different Ar pressures. The aforementioned Al (200) defect peak at 2θ = 44.9° (asterisk) is seen in the samples grown in Ar pressures up to 12 mTorr but is absent in all samples grown above 12 mTorr. Figure 4b shows X-ray reflection curves (XRR) for the samples grown at different pressures. The samples grown at 14.5 and 15 mTorr feature more extensive Kiessig thickness fringes than the others, indicating atomically abrupt substrate-metal and metal-vacuum interfaces. XRR fringes indicate a film thickness of 39 ± 1 nm. The rocking curve taken on the sample grown at 15 mTorr gives a FWHM of 0.038° at the Al(111) peak (Fig. 4b, inset). This value is the best in this study and is comparable to the rocking curve FWHM of 0.032° for the sapphire substrate used for the film. This substrate-limited rocking curve suggests that film quality has reached the point of being limited by substrate quality. In addition, AFM images (Fig. 4c) indicate that their surface roughness follows the same trend suggested by XRR, with 15 mTorr being the optimal growth pressure for producing the lowest surface roughness and smallest pinhole density.

Further improvement to film quality were achieved through improvement in substrate quality. Standard Radio Corporation of America (RCA) cleaning^37,38^, standalone acid (HCl, HNO_3_) etches, substrate annealing in air above 700 °C, or use of premium quality sapphire substrates were each attempted but yielded no significant improvement in surface roughness or crystal quality over as-received substrates. In particular, films grown after acid etches were of significantly poorer quality. We find improvement of film quality by introduction of oxygen partial pressure during the prebake, which likely repairs surface oxygen vacancies that occur in an O_2_-poor prebake process and serve as nucleation sites for defects. The pre-sputtering time of aluminum targets was made sufficiently long to remove surface oxides from the target and avoid partial oxidation of the films during growth.

**Figure 5:**
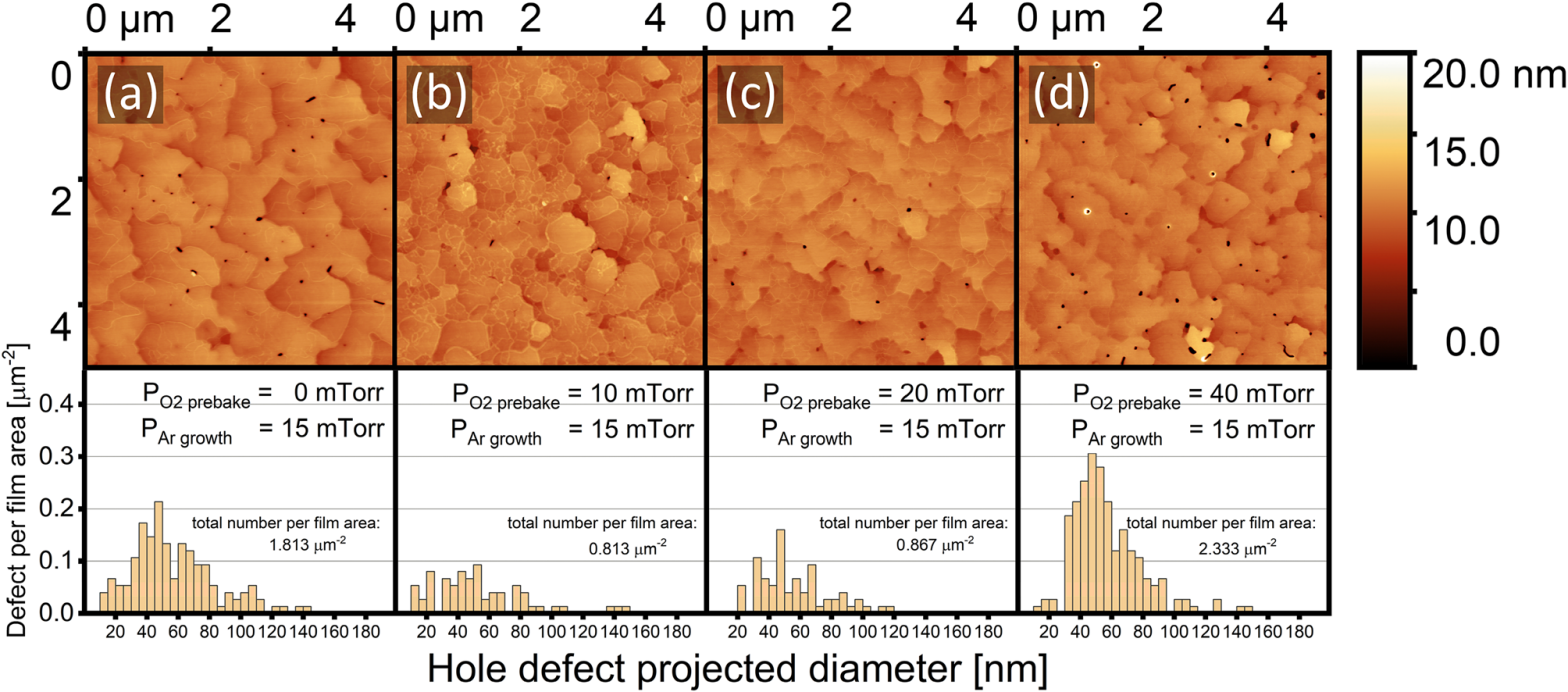
AFM images and pinhole statistics of Al/Al_2_O_3_ with substrate O_2_ prebake pressure of: (**a**) without O_2_ prebake, (**b**) 10 mTorr, (**c**) 20 mTorr, and (**d**) 40 mTorr. Statistics are surveyed over three different AFM images i.e. 75 μm^2^ for each sample, and data with pit depth less than 5 nm are treated as false positives and subsequently trimmed.

Figure 5 shows AFM images and associated pit area vs pit depth graphs for samples prebaked in O_2_ pressures of 0, 10, 20, and 40 mTorr. The large hexagonal and triangular voids commonly mentioned in the literature were not found in any film in this series. Instead, we observed hole-like defects of smaller areas and varied depths.

**Figure 6:**
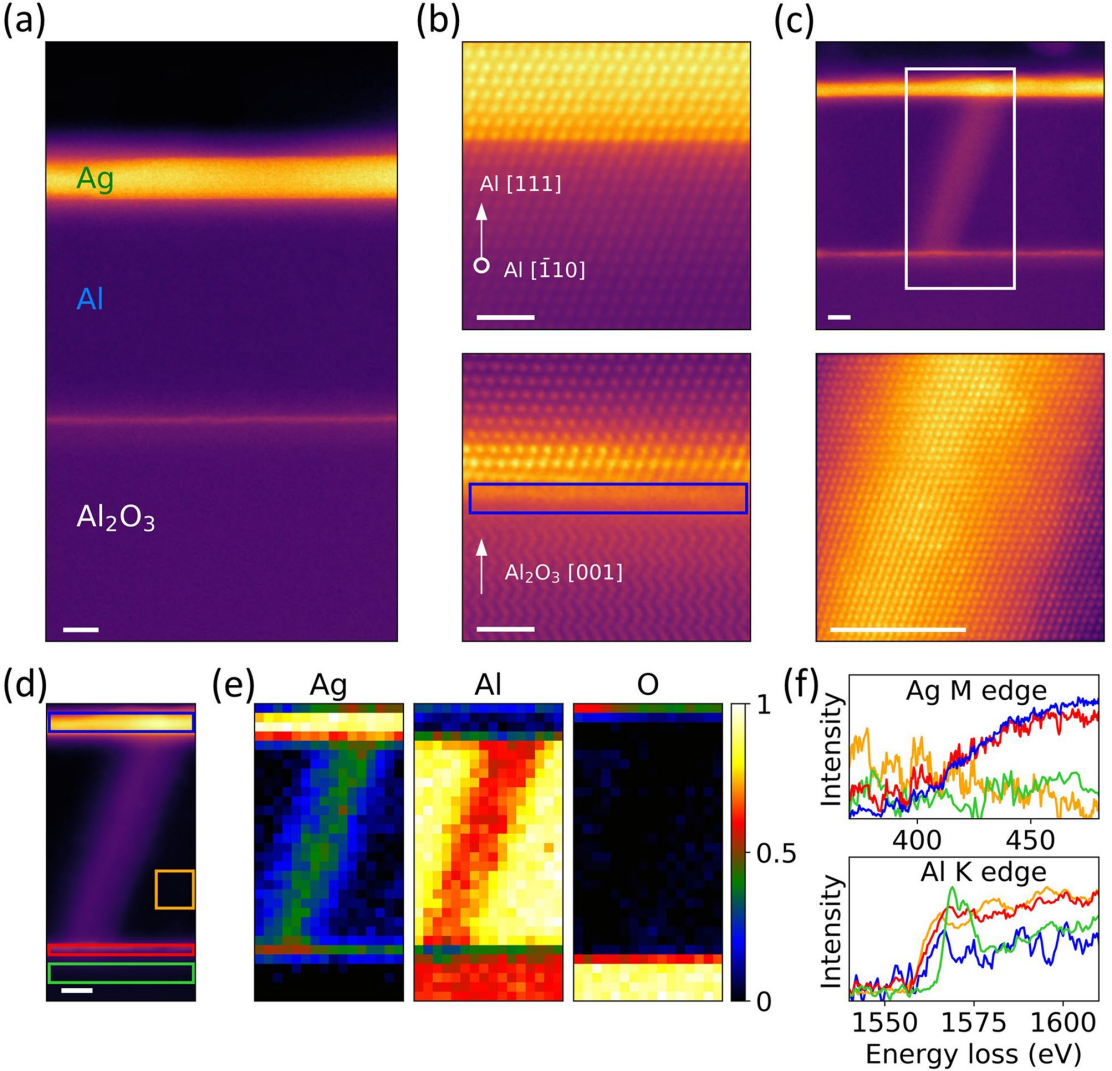
(**a**) Wide field-of-view HAADF image showing the Ag capping layer, Al thin film and sapphire substrate. (**b**) Atomic resolution HAADF image showing epitaxial Ag-capping layer on top of Al thin film (top) and Al thin film on c-plane of sapphire (bottom). The blue box at the Al/Al_2_O_3_ interface highlights the region of disorder. (**c**) Wide field-of-view (top) and atomic resolution (bottom) HAADF images showing a typical pinhole in Al thin film grown at 15 mTorr and 200 °C. The white box in (**c**) (top) shows the region chosen for EELS data acquisition. (**d**) HAADF image acquired simultaneously during EELS data acquisition. A 16 × 16 sub-pixel scanning was enabled during acquisition. (**e**) Elemental maps of Ag M edge, Al K edge and O K edge for the region highlighted in (**c**). Each elemental map is normalized within itself. (**f**) Extracted EEL spectra for Ag M and Al K edge, where the color of each spectrum corresponds to the region of same color highlighted as boxes in (**d**). Scale bars correspond to 4 nm for (**a**, **c**, **d**) and 1 nm for (**b**).

Figure 6a shows a high-angle annular dark field (HAADF) image of and Al thin film on sapphire substrate, with epitaxial Ag capping layer to fill and accentuate pinholes via contrast. For the film imaged in all of Fig. 6, a Ag capping layer of 2 nm was deposited in order to slow down oxidation and to improve imaging near the surface. Ag was chosen because it grows epitaxially and conformally on Al. Figure 6b shows atomic resolution HAADF images of Ag/Al (top) and Al/Al_2_O_3_ (bottom) interfaces. The Al thin film is projected along $(\overline{1}10)$ with surface normal oriented along [111]. The Ag/Al interface is epitaxial with Ag (higher intensity) and Al (lower intensity) atomic columns clearly distinguishable [Fig. 6b (top)]. This is because the intensity in a HAADF image is approximately proportional to the squared atomic number (~ Z^2^)^39^. The Al/Al_2_O_3_ interface, however, has a two-atomic-plane thick region of atomic disorder, which is highlighted using a blue box in Fig. 6b (bottom).

Figure 6c (top) shows a typical pinhole that we observe, with a uniform diameter of ~ 8 nm propagating through the Al film. As shown in the atomic resolution HAADF image in Fig. 6c (bottom), the thin film retains its crystal structure without the formation of any planar defects as a result of the pinhole. Figure 6d shows a simultaneously acquired HAADF image during EELS data acquisition. Elemental maps obtained from Ag M edge, Al K edge and O K edge are shown in Fig. 6e. As observed in the elemental map of Ag in Fig. 6e and the HAADF intensity in Fig. 6c, the pinhole is partially filled up by Ag from the capping layer. We also observe a 2–3 atom thick Ag layer at the weakly strained Al/Al_2_O_3_ interface. As Ag has very low solubility in Al and thus will not linger in the film bulk, thermal diffusion of Ag from pinholes and along the interface will relieve strain and is the likely mechanism behind the interfacial layer^40,41^. We observe the Ag layer at the interface to be present throughout the characterized regions of the film, even hundreds of nanometers away from a pinhole. The Ag layer does not appear to disrupt the epitaxial film structurally, but whether the Ag layer is the source of the two-atomic-plane thick region of relative disorder at the interface is unclear. We observe minimal oxidation of the thin film, as shown in O K-edge map in Fig. 6e. The extracted EEL spectra for Ag M and Al K edge are shown in Fig. 6f. The color of each spectrum corresponds to that of the region of same color highlighted in Fig. 6d. The Ag M edge has a delayed onset, which makes the Ag M edge map appear noisy.

**Figure 7:**
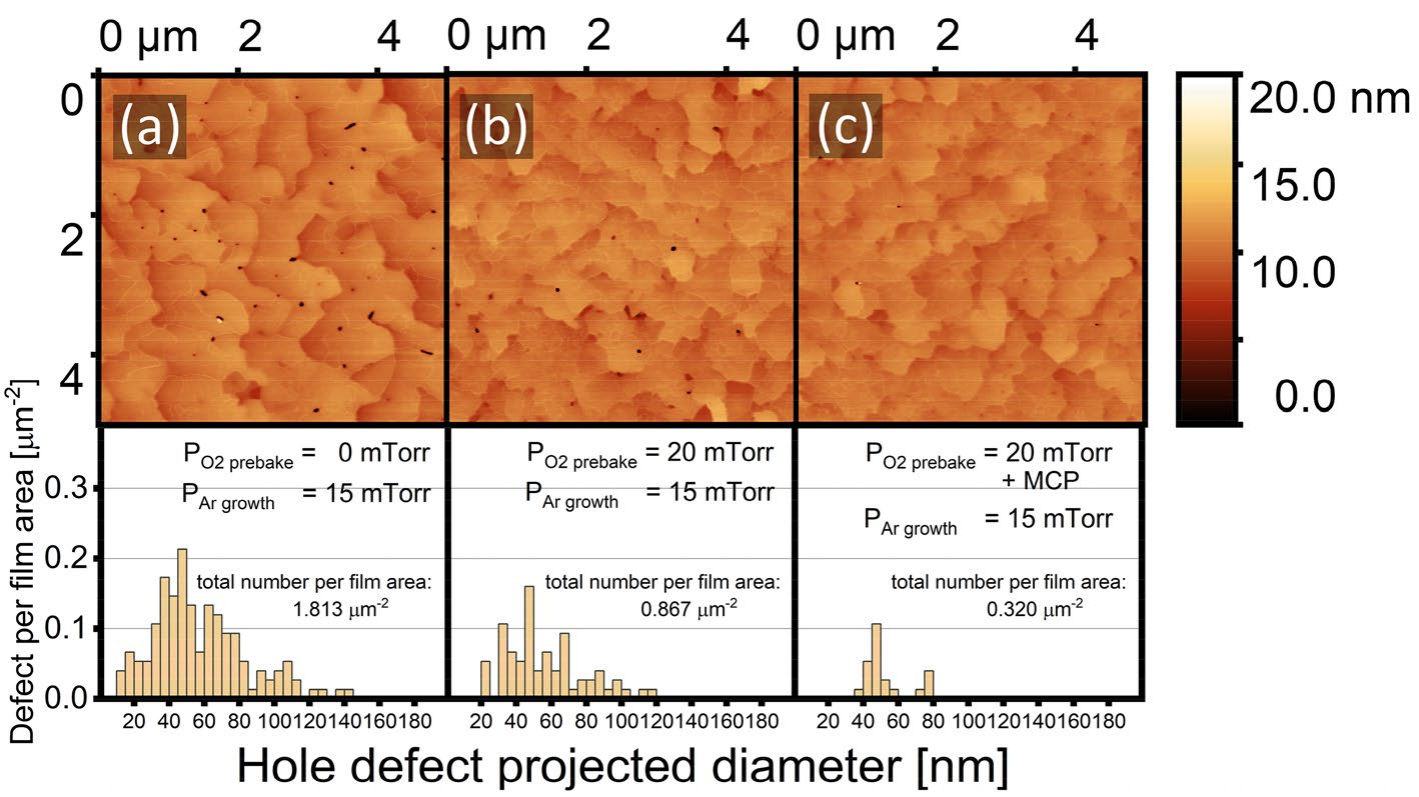
AFM images and pinhole statistics of Al/Al_2_O_3_ with substrate O_2_ prebake pressure of: (**a**) without O_2_ prebake, (**b**) 20 mTorr, and (**c**) 20 mTorr with Modified Cleaning Procedure (MCP) Statistics are surveyed over three different AFM images i.e. 75 μm^2^ for each sample, and data with pit depth less than 5 nm are treated as false positives and subsequently trimmed.

**Figure 8:**
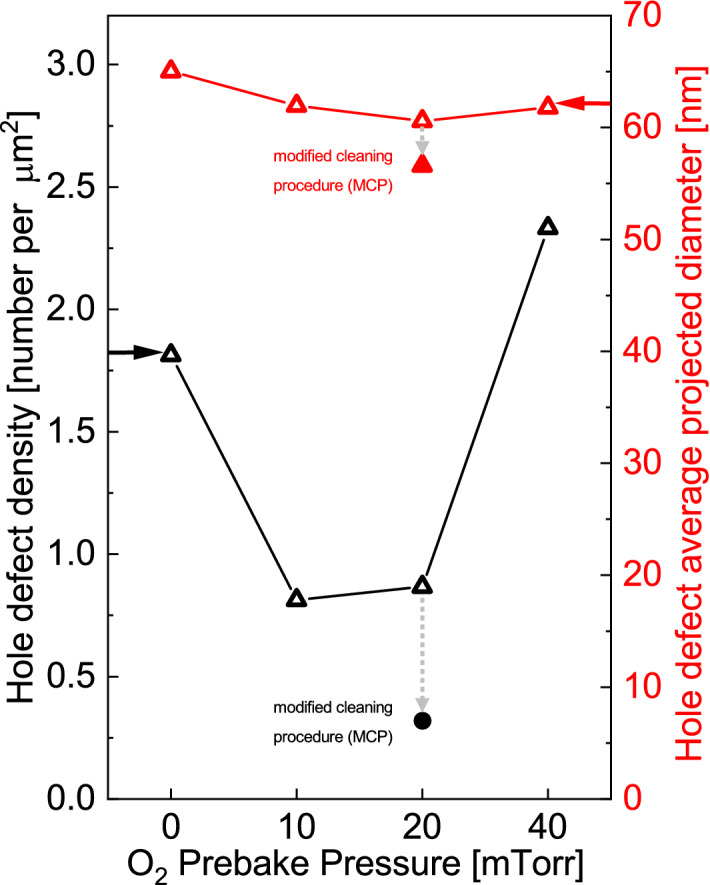
Surface density (solid black circles) and average depth (open red triangles) of hole defects for all samples, using statistics surveyed from AFM images in Figs. 6 and 7.

As pinholes in epitaxial Al propagate at an angle from the surface normal, the measured depth will be limited by the vertical orientation of the AFM’s cantilever measurement. In addition, the angle of propagation of the pinhole is 20˚ with respect to the surface normal [111] (Fig. 6c), which is also the exact angle between (111) and (101) planes when projected on the $(\overline{1}10)$ plane. This suggests that the angle of pinhole propagation is strongly dependent on the crystallographic orientation of the film. Therefore, all comparisons of film surface quality in the following discussions of Figs. 5, 7 and 8 will be consistent and useful, even though we do not yet completely understand the mechanism by which these pinholes are formed.

The graphical analysis featured above the AFM images in Fig. 5 was performed using the Gwyddion AFM image processing software, which allows processing of the images into statistically relevant quantities^42^. Substrate prebake in vacuum resulted in a film with about 1.8 hole defects per μm^2^. Substrate prebake with PO_2_ = 10 mTorr led to films with 0.8 hole defects per μm^2^, the fewest number of point defects within this sample group. However, prominent step terrace features were found for films prebaked with PO_2_ = 20 mTorr, with only minimal increase in defect number. Since step terrace features are a common indicator of thin film crystallinity and 2-dimensional growth, qualities that entail high compatibility with heterostructure fabrication, the option of further optimizing the 20 mTorr oxygen process was pursued.

At higher oxygen pressures (40 mTorr), hole defect density rises to a level that is comparable or higher than that of films grown after a substrate prebake in vacuum. Substrate baking in an oxygen-rich environment may detrimentally alter the substrate surface or exacerbate the presence of organic contaminants. It is exceedingly unlikely that over-oxidation of the Al_2_O_3_ structure will occur in our pregrowth conditions^43^, leaving improved substrate preparation as our remaining recourse. As the standard RCA procedure did not improve pinhole density or film quality in general, we have developed a modified cleaning procedure (MCP) which takes more time but proves effective in removing organic contaminant removal.

The cleaning procedure was as follows: (1) soaked in ethanol for 12 h at room temperature; (2) rinsed in deionized (DI) water and blow dried with N_2_ gas; (3) sonicated in detergent solution (detergent + ethanol + DI water 1:20:79) for 30 min at room temperature; (4) rinsed in DI water and blow dried with N_2_ gas; (5) rinsed in DI water and blow dried with N_2_ gas. Note that no acids or bases are used as compared to RCA cleans, and the preparation by soaking in DI water is replaced by a long soak in ethanol.

Figure 7 shows AFM images and associated hole defect densities for (1) a control sample that received neither oxygen infusion during substrate prebake nor the MCP, (2) a secondary control that received oxygen infusion only, and (3) the test sample that received both oxygen infusion and the MCP. The film prebaked in P_O2_ = 20 mTorr atmosphere after receiving the MCP showed superior smoothness and significantly fewer pinholes than any film that did not undergo that substrate cleaning procedure. Further investigation on many areas of the sample showed the AFM image reported in Fig. 6c to be typical. Figure 8 gives a brief review of the hole defect density and average projected diameter vs. infused O_2_ pressure. An O_2_ pressure of 20 mTorr after MCP led to both the lowest hole density and the smallest average pinholes.

Our MCP thus improves surface quality of c-plane sapphire substrates more effectively than the original RCA procedure, and may be attractive for several reasons. First, since conventional cleaning methods for sapphire substrate includes acids, including standard RCA or alternative methods using a mixture of H_3_PO_4_ and H_2_SO_4_^44^, our procedure is a great alternative for device-fabrication applications where the removal of substrate material is unacceptable. While the etch rate for H_3_PO_4_ : H_2_SO_4_ varies on different crystallographic planes of sapphire, a method to avoid acid holds great promise for application on patterned sapphire substrates (PSS). We believe our modified procedure will avoid severe distortion to the substrate surface patterning^45,46^. Lastly, our modified procedure is phosphorus-free, sulphur-free, and hydrofluoric acid (HF)-free, which bypasses some of the environmental problems of conventional industrial substrate cleaning procedures.

## Conclusions

Growth conditions to optimize crystallinity and crystal uniformity were assessed and optimized to 200 °C at 15 mTorr, with a substrate prebake at 700 °C and 20 mTorr O_2_ pressure. Films are greatly improved moreover by preparing the substrate with a MCP that nearly eliminates pinholes. The resultant film exhibited prominent Laue oscillations and XRR thickness fringes, featured no Al (200) defect XRD peak at 44.9°, displayed well-defined step-like terrace features with reduced number of surface pinholes, and exhibited reduced pinhole depth and size. We believe that our MCP, in addition to improving standard sapphire wafer surface quality, is also relatively environmentally friendly and is also likely to be more compatible with patterned surface substrates than standard acid etches.
